# Supplementary material for: An inducible CRISPR-ON system for controllable gene activation in human pluripotent stem cells
Source: Protein Cell. 2017 Jan 23;8(5):379–93. doi: 10.1007/s13238-016-0360-8 (PMC5413595; doi:10.1007/s13238-016-0360-8)

Figure S1. Activating exogenous genes through dCas9-VPR system in mESCs and MEFs

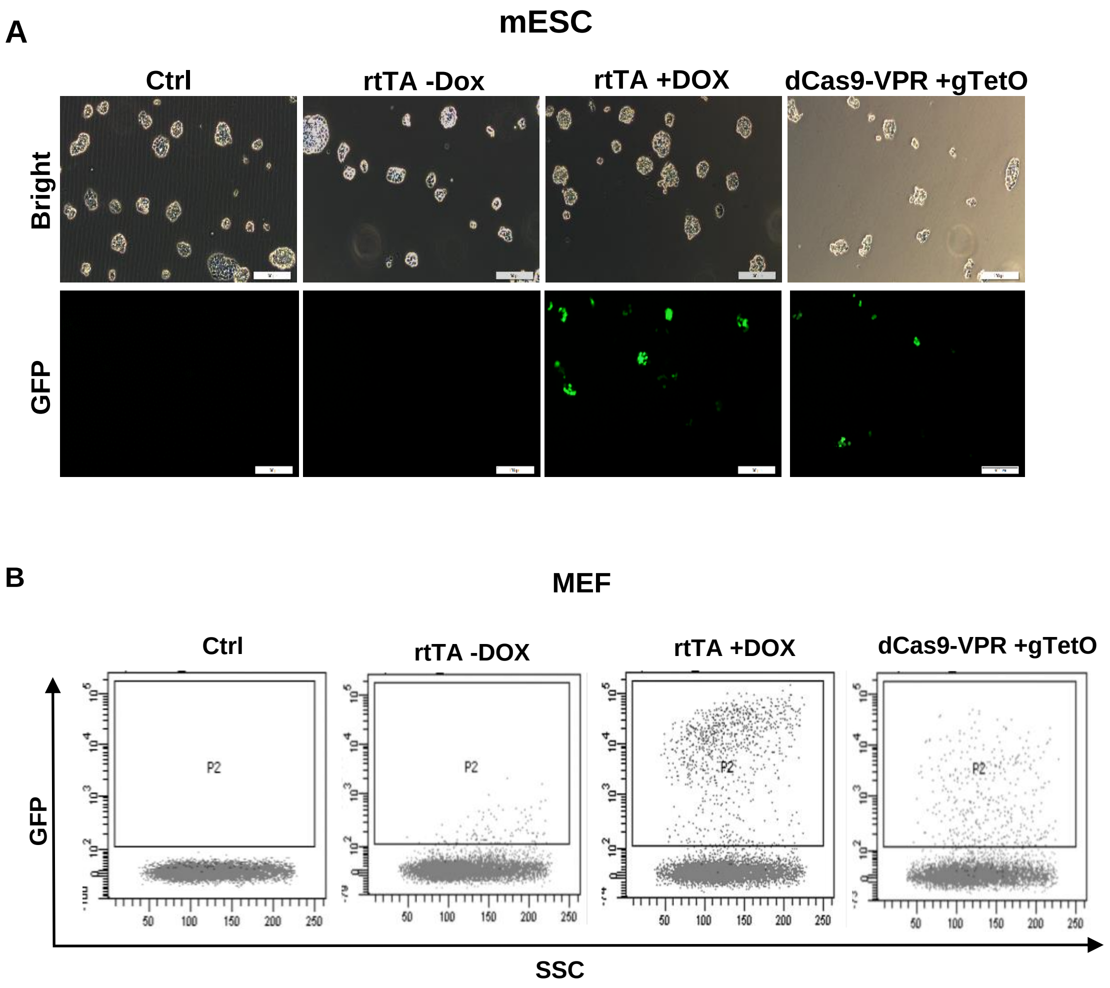

Figure S2. Characterization of iVPR hESC clones

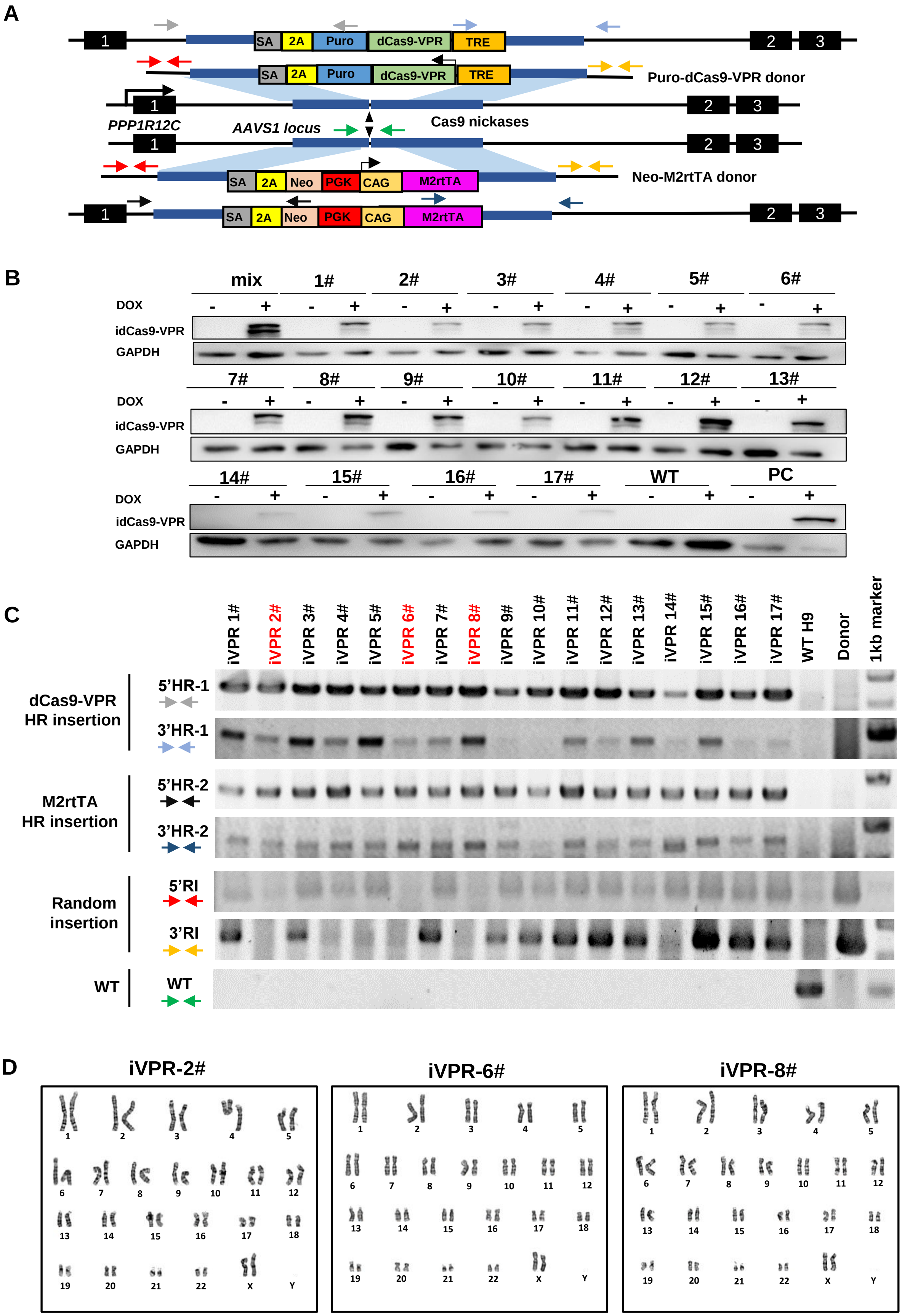

Figure S3. Upregulation of *NANOG* by dCas9-VPR promoted naïve state of pluripotency

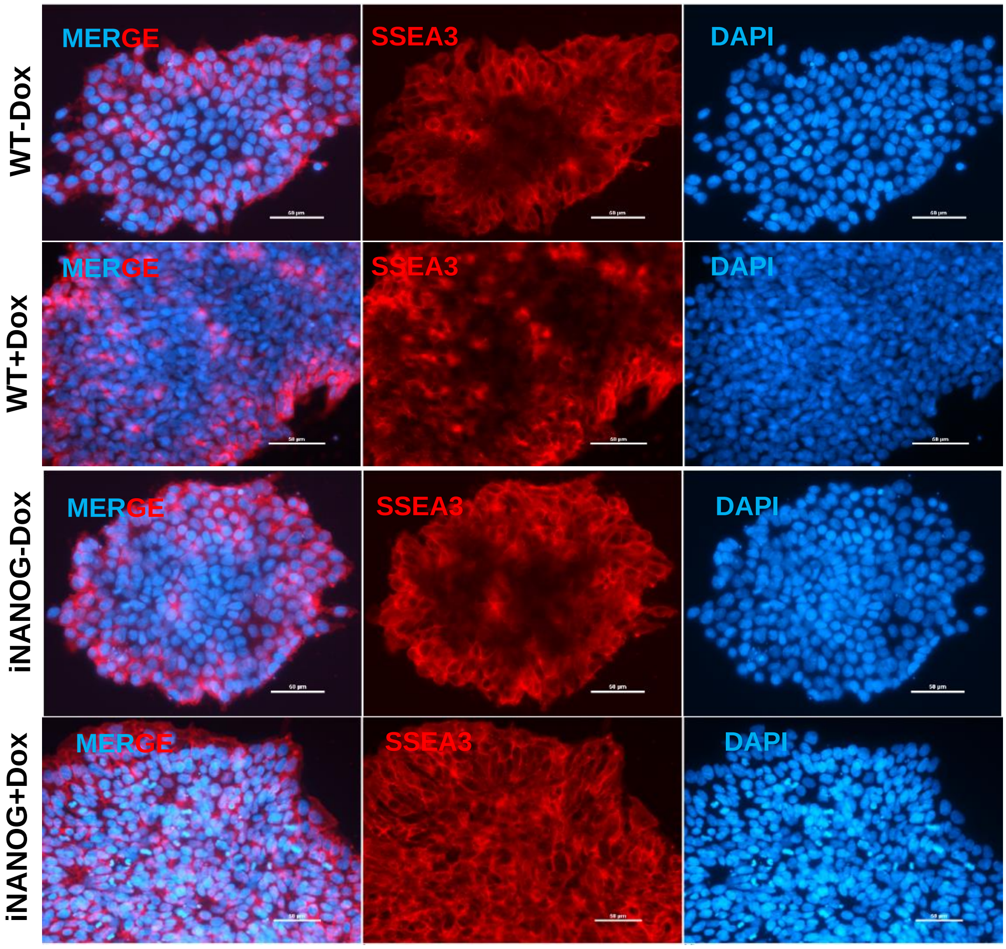

Figure S4. Analysis of iNANOG cells integration in mouse blastocysts

A

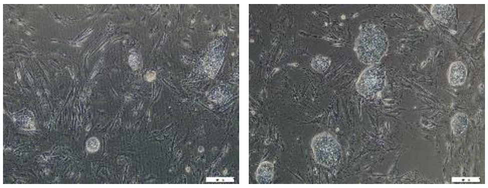

B

The number of blastocysts with GFP^{+} hESCs

Hours	iNANOG +Dox 2iL/FK	iNANOG +Dox	iNANOG -Dox	WTSG +Dox
0	51 (100%)	33 (100%)	59 (100%)	51 (100%)
1	50 (98%)	32 (97%)	58 (98%)	50 (98%)
2	50 (98%)	32 (97%)	58 (98%)	49 (96%)
3	49 (96%)	32 (97%)	57 (97%)	48 (94%)
4	48 (94%)	31 (94%)	56 (95%)	45 (88%)
5	46 (90%)	30 (91%)	56 (95%)	43 (84%)
6	46 (90%)	30 (91%)	53 (90%)	43 (84%)
7	46 (90%)	30 (91%)	46 (78%)	41 (80%)
8	45 (88%)	29 (88%)	44 (75%)	39 (76%)
9	45 (88%)	29 (88%)	42 (71%)	38 (75%)
10	43 (84%)	26 (79%)	34 (58%)	37 (73%)
11	43 (84%)	26 (79%)	32 (54%)	37 (73%)
12	42 (82%)	24 (73%)	29 (49%)	31 (61%)
13	42 (82%)	24 (73%)	29 (49%)	31 (61%)

C

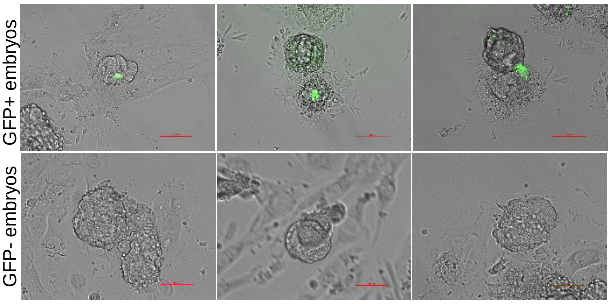

D

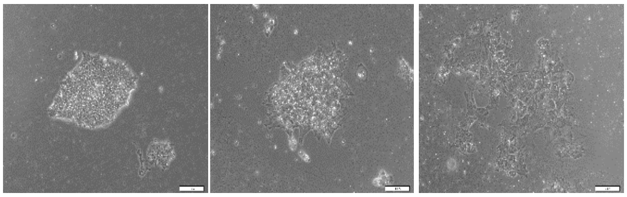

Supplement: Supplementary file 1 — Supplementary material 1 (PDF 879 kb) [file 13238_2016_360_MOESM1_ESM.pdf]
