# Supplementary material for: An inducible CRISPR-ON system for controllable gene activation in human pluripotent stem cells
Source: Protein Cell. 2017 Jan 23;8(5):379–93. doi: 10.1007/s13238-016-0360-8 (PMC5413595; doi:10.1007/s13238-016-0360-8)
Supplement: Supplementary file 2 — Supplementary material 2 (PDF 245 kb) [file 13238_2016_360_MOESM2_ESM.pdf]

Supplemental Information

An inducible CRISPR-ON system for controllable gene activation in human pluripotent stem cells

Jianying Guo, Dacheng Ma, Rujin Huang, Jia Ming, Min Ye, KehKooi Kee, Zhen Xie and Jie Na

Supplemental Figure Legends

Figure S1. Activating exogenous genes through dCas9-VPR system in mESCs and MEFs

(A) MESCs were co-transfected with the reporter plasmid containing GFP driven by the TRE promoter together with either CAG-rtTA plasmid, or with dCas9-VPR and gTetO plasmids. Scale bar, 100 μm. Clear GFP fluorescence can be observed in rtTA plus Dox treatment group, but not without Dox. DCas9-VPR plus gTetO group also showed strong GFP fluorescence.

(B) MEF cells were co-transfected with same series of plasmids as in (A). Cells were harvested 3 days after transfection and analyzed using flow cytometry. The groups and treatment were indicated on top of each graph. Similar results were obtained, both rtTA plus Dox treatment group and dCas9-VPR plus gTetO group had obvious GFP positive population.

Figure S2. Characterization of iVPR hESCs

(A) Schematic view of *AAVS1* targeting plasmids and strategy. Colorized arrowheads indicate primers for *AAVS1* insertion confirmation by genomic DNA PCR. Donor plasmids: Puro-dCas9-VPR donor, TRE promoter driving dCas9-VPR followed by puromycin resistance gene (Puro); 2A, self-cleaving peptide 2A; SA, splice acceptor. Neo-M2trTA donor, M2rtTA was driven by a CAG promoter; Neomycin resistance gene (Neo) was driven by a PGK promoter.

(B) Western blot showing that 17 single clones can all express dCas9-VPR upon Dox addition. DCas9-VPR constitutively expression H9 cell line was used as the positive control (PC).

(C) Genomic DNA PCR analysis of correct *AAVS1* locus insertion. DCas9-VPR homology recombination in one of the *AAVS1* allele (HR-1), M2rtTA homology recombination in another *AAVS1* allele (HR-2), random insertion (RI), and the wild type *AAVS1* alleles (WT). Correct targeted clones were highlighted in red.

(C) Karyotype of iVPR hESC clone 2#, 6# and 8#. All three single clones show a normal karyotype with 46, XX.

Figure S3. Upregulation of NANOG by dCas9-VPR promoted naïve state of pluripotency

Immunostaining result shows that compared to wild type H9 control, upregulation of *NANOG* by dCas9-VPR system lead to elevated and more homogeneous SSEA3 expression. Scale bar, 50 μm.

Figure S4. Analysis of iNANOG cells integration in mouse blastocysts

(A) Naïve cultured iNANOG cells shows bigger colonies upon *NANOG* induction. Scale bar, 100 μm.

(B) Table listing the number of blastocysts with GFP^+ hESCs from 0 hours to 13 hours.

(C) Representative images of attached embryos with or without iNANOG cells after 3 days of *in vitro* culture in IVC1 medium. 3 out of 40 injected embryos showed GFP^+ cells within the outgrowth mouse ICM cells. Scale bar, 100 μm.

(D) HESC cultured in IVC1 or IVC2 medium for 3 days showed poor proliferation and differentiated morphology compared to their counterpart cultured in E8 medium. Scale bar, 50 μm.

Video Legends

Movie S1. Time-lapse video of 2iL/FK cultured, Dox induced iNANOG cells interaction with cells inside mouse blastocysts. Related to Figure 6.

Movie S2. 3D reconstruction of confocal images of iNANOG cells integrates into the ICM of E4.5 mouse blastocyst. Related to Figure 6.

Movie S3. 3D reconstruction of confocal images of iNANOG cells integrates into the TE region of E4.5 mouse blastocyst. Related to Figure 6.

Movie S4. 3D reconstruction of confocal images of iNANOG cells integrates into both the ICM and TE region of E4.5 mouse blastocyst. Related to Figure 6.

Movie S5. 3D reconstruction of confocal images of an E4.5 mouse blastocyst without iNANOG cells. Related to Figure 6.

Supplemental Tables

Table S1. Oligonucleotides used in this study

Oligonucleotides for generating gRNA targeting the promoter regions of interest

	Forward	Reverse
**TRE gRNA**	5' TACGTTCTCTATCACTGATA 3'	5' TATCAGTGATAGAGAACGTA 3'
**NANOG gRNA1**	5' GAATATGGTTCAACAGGAAT 3'	5' ATTCCTGTTGAACCATATTC 3'
**NANOG gRNA2**	5' GCAGAGTAACCCAGACTAGG 3'	5' CCTAGTCTGGGTTACTCTGC 3'
**HOXA9 gRNA1**	5' ATCACTCCGCACGCTATTAA 3'	5' TTAATAGCGTGCGGAGTGAT 3'
**HOXA9 gRNA2**	5' GCGTTATTGTTCTGCTGGAC 3'	5' GTCCAGCAGAACAATAACGC 3'
**HOXA10 gRNA1**	5' AGGTGTCGGGGTGCGTCTCC 3'	5' GGAGACGCACCCCGACACCT 3'
**HOXA10 gRNA2**	5' ATATCAATCGCGGGCTCTGA 3'	5' TCAGAGCCCGCGATTGATAT 3'
**SNAIL1 gRNA1**	5' CCGAAGGCCACTCCCCGAGC 3'	5' GCTCGGGGAGTGGCCTTCGG 3'
**SNAIL1 gRNA2**	5' CGAGCCTCCGATTGGCGCGG 3'	5' CCGCGCCAATCGGAGGCTCG 3'
**MESP1 gRNA1**	5' CAGTCAAGGGGCTGGCACCT 3'	5' AGGTGCCAGCCCCTTGACTG 3'
**MESP1 gRNA2**	5' CCCGGGCTTCACCCAACACT 3'	5' AGTGTTGGGTGAAGCCCGGG 3'
**GATA5 gRNA1**	5' AACGGTCCTGAGGGCCTGCC 3'	5' GGCAGGCCCTCAGGACCGTT 3'
**GATA5 gRNA2**	5' GCCTTTCCCCCCGCGGATTT 3'	5' AAATCCGCGGGGGGAAAGGC 3'

Oligonucleotides for generating *AAVS1* locus knock-in gRNA

	Forward	Reverse
***AAVS1* gRNA up**	5'GTCCCTAGTGGCCCCACTGT3'	5'ACAGTGGGGCCACTAGGGAC3'
***AAVS1* gRNA down**	5'GACAGAAAAGCCCCATCCTT3'	5'AAGGATGGGGCTTTTCTGTC3'

Table S2. PCR primers used in this study

PCR primers for generating southern blot probes

	Forward	Reverse	Probe size
**EXT-probe**	5' ACAGGTACCATGTGGGGTTC 3'	5' CTTGCCTCACCTGGCGATAT 3'	445 bp
**INT-probe**	5' AGGTTCCGTCTTCCTCCACT 3'	5' GTCCAGGCAAAGAAAGCAAG 3'	388 bp

PCR primers for genomic DNA PCR to verify idCas9-VPR *AAVS1* locus knock-in.

	Forward	Reverse	Fragment size
**5'RI**	5' TCCCTTTAGTGAGGGTTAATTCTGA 3'	5' GTTCTTTCCTGCGTTATCCCCT 3'	384 bp
**3'RI**	5' GTGAACTTTACCCGGTGGTGC 3'	5' GCGTGCAATCCATCTTGTTCA 3'	538 bp
**WT**	5' CTCCATCGTAAGCAAACCTTAGAG 3'	5' CCTGTGGATTCGGGTCACCT 3'	475 bp
**3'HR-1**	5' CCTGGGATACCCCGAAGAGT 3'	5' GCGATCTGACGGTTCACTAAAC 3'	1369 bp
**5'HR-1**	5' CGTGGGCTTGTACTCGGTCA 3'	5' CTCCCATAGCTCAGTCTGGTCTATC 3'	1147 bp
**5'HR-2**	5' CTCCCATAGCTCAGTCTGGTCTATC 3'	5' GACAGGTCGGTCTTGACAAAAAG 3'	1302 bp
**3'HR-2**	5' TACAAATGTGGTATGGCTGATTATG 3'	5' CCCGAAGAGTGAGTTTGCC 3'	1279 bp

Q-PCR primers used in this study

Gene	Accession No.	Forward primer	Reverse primer	Tm.
AFP	NM_001134	AGAACCTGTCACAAGCTGTG	GACAGCAAGCTGAGGATGTC	60
dCas9-VPR	----	AACTGGAAAATGGCAGGAAGAG	GTCAATCCTTGTTTCGTAGAGTCCT	60
GAPDH	NM_002046	TGATGACATCAAGAAGGTGGTGAAG	TCCTTGGAGGCCATGTGGGCCAT	60
GATA5	NM_080473	ACAGCTCAGCAGCCACTTCG	CCGTGGAGGGGAAGGCAAAG	60
GDF3	NM_020634	AGACTTATGCTACGTAAAGGAGCT	CTTTGATGGCAGACAGGTTAAAGTA	60
LEFTYB	NM_020997	TGCTACAGGTGTCGGTGCAGAGG	AGAAACGGCCACTTGAAGGCCAGG	60
NANOG	NM_024865	GGATGGTCTCGATCTCCTGA	CCTCCCAATCCCAAACAATA	60
PRDM14	NM_024504	AATCATTGGTGGCGACAACGA	CCCGTACAGAACGAAGTGCAG	60
SNAIL1	NM_005985	TGTCTGCGTGGGTTTTTGTA	GGGGGTGGATTATTGCATAG	60
OCT4	NM_002701	CGACCATCTGCCGCTTTGAG	CCCCCTGTCCCCCATTCCTA	60
SOX2	NM_003106	CCCCCGGCGGCAATAGCA	TCGGCGCCGGGGAGATACAT	60
CDX2	NM_001265	CCTCCGCTGGGCTTCATTCC	TGGGGGTTCTGCAGTCTTTGGTC	60
PAX6	NM_000280	ACTGGGGAAGGAATGGACTT	AATTCGTGGCAAAGCTTGTT	60
SOX1	NM_005986	GGGACCTTGAGGGTTTTCTC	GGCCCACATCCTAATCTTGA	60
MESP1	NM_018670	GCTCTGTTGGAGACCTGGAT	GTCTGCCAAGGAACCACTTC	60
HOXA9	NM_152739	CAGTTCCAGGGTCTGGTGTT	GCGCCTTCTCTGAAAACAAT	60
HOXA10	NM_018951	CCTTCTCCAGCTCCAGTGTC	CGAGAGCAGCAAAGCCTC	60

Supplemental Experimental Procedures

Generation and characterization of iVPR knocking-in cell line

The iVPR *AAVS1* knock-in cell line was generated by the CRISPR method. Tet-On system were obtained from Clontech. PiggyBac plasmids were generous gift from the Sanger institute, Cambridge, UK. The homologous recombination donor vectors for *AAVS1* locus targeting was a generous gift from Dr Danwei Huangfu (Gonzalez et al., 2014). Cas9 nickase, *AAVS1* gRNAs and homologous recombineering donor plasmids were transfected into H9 cells using Neon microporator (Invitrogen) following manufacturer's instructions. Cells were plated on DR4 MEF feeders in hESC medium supplemented with 10 μM Y-27632 for the first day. After puromycin and neomycine double selection for 14 days, single colonies were picked and expanded. Genomic DNA PCR was performed to identify clones with correct *AAVS1* locus targeting. Correct targeted iVPR clones were further confirmed by southern blot. For 5' internal probe hybridization, Sph I was used to digest genome DNA; for 3' external probe hybridization, Bgl II was used to digest genomic DNA. DNA Molecular Weight Marker II, Digoxigenin-labeled (Roche 11218590910) were used as marker. The primer sequences were listed in table S2.

Karyotype analysis

Karyotyping was performed at the Reproductive Medicine Center of the Peking University 3rd Hospital. For each iVPR clone, 20 metaphase chromosome spreads were imaged and analyzed.
